# Adaptation, Transformation and Resilience in Healthcare: Comment on "Government Actions and Their Relation to Resilience in Healthcare During the COVID-19 Pandemic in New South Wales, Australia and Ontario, Canada"

**DOI:** 10.34172/ijhpm.2022.7043

**Published:** 2022-02-28

**Authors:** David G. Angeler, Harris A. Eyre, Michael Berk, Craig R. Allen, William Hynes, Igor Linkov

**Affiliations:** ^1^Swedish University of Agricultural Sciences, Department of Aquatic Sciences and Assessment, Uppsala, Sweden.; ^2^The PRODEO Institute, San Francisco, CA, USA.; ^3^IMPACT, The Institute for Mental and Physical Health and Clinical Translation, Deakin University, Geelong, VIC, Australia.; ^4^Global Brain Health Institute, University of California, San Francisco (UCSF), San Francisco, CA, USA.; ^5^Trinity College Dublin, Dublin 2, Ireland.; ^6^Department of Psychiatry and Behavioral Sciences, Baylor College of Medicine, Houston, TX, USA.; ^7^Neurosience-inspired Policy Initiative, Organisation for Economic Co-Operation and Development (OECD), Paris, France.; ^8^Meadows Mental Health Policy Institute, Dallas, TX, USA.; ^9^Department of Psychiatry, University of Melbourne, Melbourne, VIC, Australia.; ^10^Orygen Youth Health, University of Melbourne, Melbourne, VIC, Australia.; ^11^The Florey Institute for Neuroscience and Mental Health, University of Melbourne, Melbourne, VIC, Australia.; ^12^Center for Resilience in Agricultural Working Landscapes, School of Natural Resources, University of Nebraska – Lincoln, Lincoln, NE, USA.; ^13^US Army Engineer Research and Development Center, Concord, MA, USA.; ^14^Carnegie Mellon University, Pittsburgh, PA, USA.

**Keywords:** Resilience, COVID-19, Healthcare Systems, Health Policy, Healthcare Management

## Abstract

Adaptive capacity is a critical component of building resilience in healthcare (RiH). Adaptive capacity comprises the ability of a system to cope with and adapt to disturbances. However, "shocks," such as the current coronavirus disease 2019 (COVID-19) pandemic, can potentially exceed critical adaptation thresholds and lead to systemic collapse. To effectively manage healthcare systems during periods of crises, both adaptive and transformative changes are necessary. This commentary discusses adaptation and transformation as two complementary, integral components of resilience and applies them to healthcare. We treat resilience as an emergent property of complex systems that accounts for multiple, often disparately distinct regimes in which multiple processes (eg, adaptation, recovery) are subsumed and operate. We argue that Convergence Mental Health and other transdisciplinary paradigms such as Brain Capital and One Health can facilitate resilience planning and management in healthcare systems.

 Smaggus and colleagues recently studied government actions in response to the coronavirus disease 2019 (COVID-19) pandemic that emanated from the severe acute respiratory syndrome coronavirus 2 (SARS-CoV-2) virus in relation to resilience in healthcare (RiH).^1^ Conducting qualitative media content analysis in Ontario (Canada) and New South Wales (Australia), they found that resilience management in terms of the need to anticipate, monitor, respond and learn from the crisis was invoked in media releases of both governments. The authors concluded that “articulating a proactive vision of resilience and recognizing the complex nature of current systems could enhance governments’ ability to coordinate resilient performance in healthcare. Reflection on how anticipation relates to resilience appears necessary at both the practical and conceptual levels to further develop the capacity for RiH.”^1^

 We concur with this assessment. However, we have identified several areas where additional clarifications may help to promote resilience planning and management in healthcare. The complex adaptive systems view of resilience needs to be discussed from the perspective of resilience as an emergent property,^2,3^ which considers multi-scale social-ecological, engineering and economic dimensions of resilience. Although this complexity is recognized in RiH, the predominating feature of resilience in RIH is adaptive capacity, a system’s capacity to deal with and recover from disturbances. This narrow view of RiH is common^6-8^ and exemplified by the definition used by Smaggus et al^1^; ie, the capacity to consistently deliver safe, high-quality healthcare through adaptations at multiple system levels in response to challenges and disruptions. This definition lacks important process-based (recovery, persistence, robustness) and systemic (transformation) aspects inherent in the resilience of complex adaptive systems.^2,3^

 It is equally necessary to account for these aspects, especially transformation, because alternative, often novel system regimes can arise when adaptive capacity is exhausted and critical disturbance thresholds surpassed. This suggests that Wood’s concepts of sustained adaptability and graceful extensibility used in RiH^7^ to create and maintain adaptive capacity may be of limited application. There is ample evidence about the ubiquity of regime changes and alternative regimes, such as lakes shifting from clear water to turbid regimes, grasslands to forests, democracies to authoritarian regimes and humans developing chronic disease. These examples make clear that ecosystems and other systems of people and nature can neither absorb disturbances infinitely, nor be perpetually forced to stay in a specific regime through management.^4,9^

 Important management implications follow from the capacity of complex adaptive systems to exist in alternative regimes. Alternative regimes are often irreversible and deleterious for human well-being and livelihoods due to limited provisioning of goods and services (eg, food, medicines, and supply chains).^10-12^ Frequently, costly and inefficient reactive management can then only mitigate but not restore degraded regimes to a more desirable regime.^4^ Also, management, even if well-intended to support human needs, can paradoxically erode adaptive capacity to an extent that a regime shift becomes inevitable.^13^ Consequently, a system flips into an alternative regime with substantially different structures and functions relative to the previous system.^3^

 System collapse and reorganization to an alternative regime often occurs when management focuses on optimizing one system variable, such as agricultural productivity. Such command-and-control management^13^ is also reminiscent of actions currently taken by many governments to handle the COVID-19 pandemic. That is, stringent measures such as repeated, prolonged lockdowns with the sole objective to protect healthcare systems against collapse may erode the broader socio-economic system, leading to potentially severe repercussions – reducing demand, employment, and social engagement while increasing mental health disorders, distrust, social polarization and revolt, and inequalities. In the information domain, misinformation, conspiracy theories, and the resulting resistance to scientific, evidence-based efforts to mitigate the crisis (anti-vaccine movement) may reinforce and aggravate the situation.

 The current COVID-19 crisis is an example of the high uncertainty associated with the complexity inherent in social-ecological processes. There is false confidence that we can control system processes, manifested by fundamental unknowns and radical uncertainty; that is, whether human-made systems and nature are genuinely able to adapt to a world stricken by unpredictable disturbances or whether collapse and the emergence of alternative regimes is inevitable. The pandemic shock was unavoidable but novel and its duration is still too short to ascertain if regime shifts have already occurred or if social-ecological systems are currently in a phase of reorganization that may eventually stabilize in a new local, regional or global social-economic-cultural-ecological system in which healthcare systems are embedded. It is also impossible to predict how the COVID-19 or similar sudden, global shocks will affect human livelihoods and well-being in the long run. Interactions with the compounded disturbances arising from climate change (for instance, food insecurity, mass migration, sea-level rise, extreme weather events) can have catastrophic consequences for humanity that may “dwarf”^14^ the impacts of the COVID-19 pandemic. Increasing evidence already points to a demise in human (mental) health because of such complex change.^11,12^

 The proverbial “new normal” suggests that regime change is at least considered implicitly in the discourse across sectors of societies since the onset of COVID-19. This implicitness is also evident in Hollnagel’s^6^ anticipation criterion of resilience used in RiH that considers probabilistic modes that extrapolate past trends to a vision of the future. This anticipation mode is implicit in that it fits the notion of single and alternative regimes or adaptation and regime change without necessarily mechanistically discerning these resilience aspects. However, Hollnagel’s realistic anticipation mode explicitly invokes transformation, the purposeful erosion of a less desirable regime to enable the emergence of a more desirable regime. It acknowledges that novel futures can differ fundamentally from the past and that such futures may pose entirely new challenges for societies.^1,6^ This anticipation mode of resilience deserves more serious consideration in RiH planning and management because it exemplifies non-stationary thinking (both the dynamics and the bounds of a system change, often non-linearly and irreversibly) opposed to a stationary, equilibrium-based perspective on complex systems change, which is inherent in adaptive capacity. It also implies that both adaptation and transformation will be required simultaneously and at different scales (eg, individuals, societies) at different times and in different subsystems (eg, economy, policy) of a social-ecological system.

 There are fundamental unknowns inherent in non-stationary change, which are pervasive, and crucial but impossible to account for, and thus often ignored in resilience, specifically transformative, management. The “unknown unknowns” form the kernel of uncertainty. They embody a knowledge domain that is beyond current human cognition yet contains “hidden solutions” that may eventually help to navigate complex situations. However, any attempt to define and operationalize what we do not know that we do not know is a “contradiction in absurdum.”^15^ This highlights that any clear prescriptive solution including for instance the creation of fail-safes and back-up arrangements necessary for resilience management are highly uncertain and likely not efficient. However, the current COVID-19 pandemic exemplifies how the political and public need for certitude runs counter to the uncertainty that governs complex systems dynamics. Assuming certitude gives a false, misleading and dangerous sense of security, which is evident in many elements of the COVID-19 crisis.

 The pandemic is a clear case highlighting the difficulty to envision, identify, and cope with societal challenges and change. Western societies that have increasingly primed individualism, materialism and linear economic growth over recent decades have so far not consistently put humanity on a path of sustainable development through goal-oriented problem solving. On the contrary, ongoing environmental challenges and intensifying social-ecological problems exemplified by an increasing mental health pandemic with unprecedented impact on societies, economies and healthcare systems continue unabated. In the face of such complexity, scenario planning to create different visions of the future is helpful. Recognizing the limitation of siloed scientific, political and economic approaches for solving vexing, wicked social-ecological problems, such as pandemics and climate change, new scientific paradigms are emerging that can facilitate scenario planning.^16^ For instance, Convergence Mental Health^17^ and Brain Capital^18^ are transdisciplinary paradigms which consider environmental, social and governance factors from the neural to cognitive to policy levels for brain health and the development of preparedness and coping/resilience mechanisms to improve mental health. These paradigms have strong potential for holistic and integral planning of a range of worst-case to best-case scenarios to navigate the contradiction absurdities inherent in complex social-ecological challenges. We advocate for transdisciplinary collaborations across cultures, sectors of societies and scientific disciplines because innovation that forms the basis for progress and problem solving takes place at the intersection of disparate expertise areas and life experiences.^19^

 It is time for societal transformation to meet sustainable development goals, especially as they pertain to human health systems. We conclude with a model that could inform scenario planning for RiH (Figure).

**Figure:**
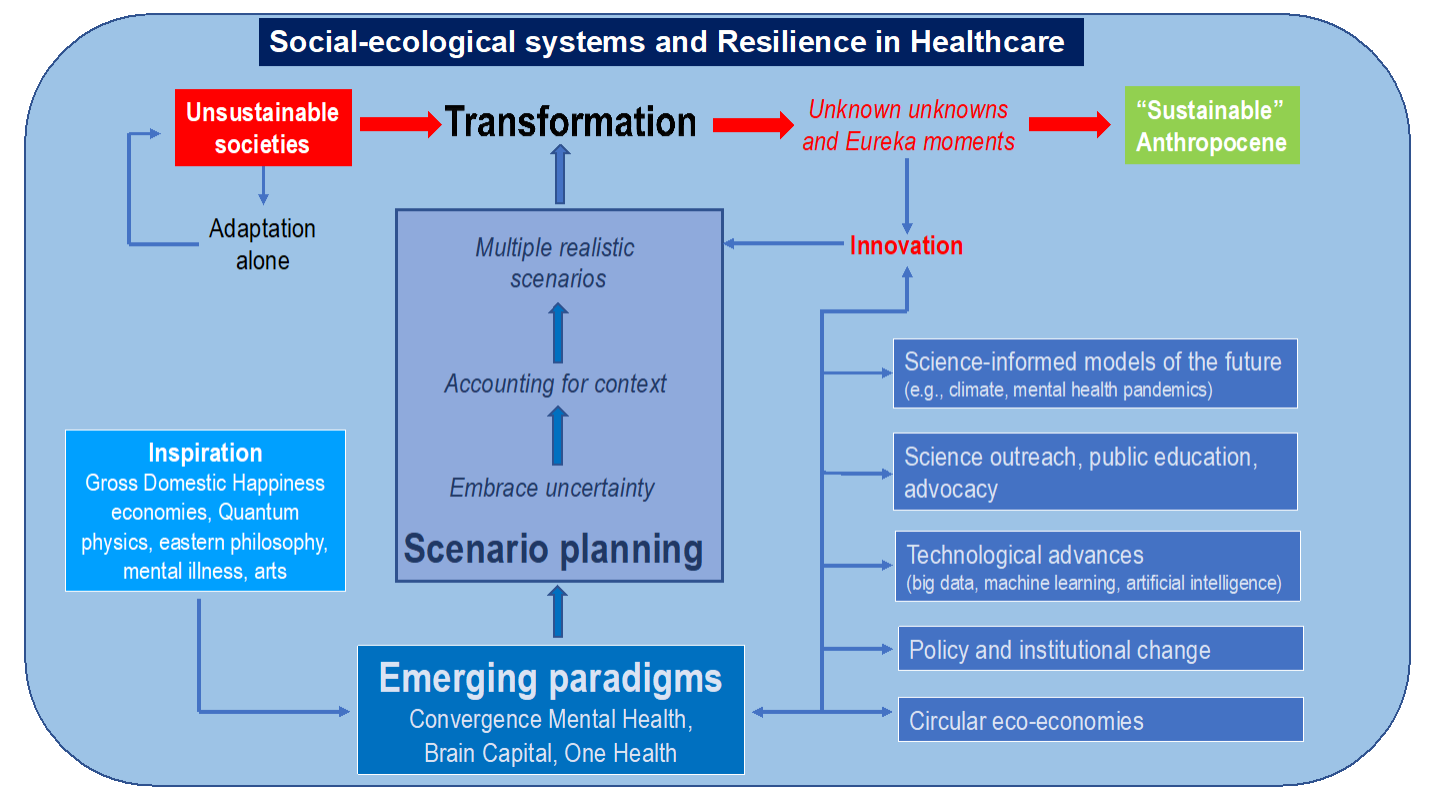
Theoretical Model Demonstrating Transformation of Unsustainable Societies and Embedded Healthcare Systems to a More Sustainable Anthropocene for Humanity Through Scenario Planning. The model is meant for demonstrating the complexity related to transformative change. The potential for transdisciplinary approaches (Convergence Mental Health, One Health) is therefore not exhaustive.

 This model suggests that realistic scenario planning needs to consider RiH as an integral but not isolated domain of complex social-ecological systems. That is, sustainable and resilient healthcare systems transformation can likely only occur with the transformation of higher system-level entities such as economies and governance. This suggests that current approaches to RiH management, despite accounting for system complexity, will be likely insufficient and ineffective for achieving sustainable and resilience healthcare systems in the long run. Resilience by design (eg, endogenous reallocation of resources in medical systems, creation of redundancies in infrastructure structure and services [ie, intensive care beds, medical equipment]) and resilience by intervention (eg, stockpiling sensitive equipment, government mandates on health and production, expertise diversification and mobility of healthcare personal) in response to a pandemic or other disturbances will need to be accompanied by transformation in social dimensions (eg, understanding of resilience by institutions and the lay public through targeted outreach and science advocacy). Also, economies moving towards more circular and biofueled models need to consider a reshaping of private and public funding of healthcare, for instance, through philanthropy, non-for-profit organizations or angel investors.^17^ RiH and more broadly social-ecological systems transformation needs to ask questions about which steps are necessary to take as a function of science-based predictive models of potential futures with novel envisioned social-ecological disturbance regimes, such as conflicts arising from resource scarcity and the consequences for healthcare of such conflicts. There is likely no one-size-fits-all approach to scenario planning given different social-ecological contexts. Consider, for instance, a relatively simple example of hurricane-prone areas where the increasing frequency and severity of extreme weather events due to climate warming puts healthcare facilities, and other urban infrastructure, at high risk. In this case, rebuilding facilities after storms seems the least resilient option. Scenarios envisioning, for instance, the construction of healthcare installations below ground may facilitate transformation towards a more resilient future. There are also more complex and challenging situation, such as the interaction of prolonged heat waves causing thermal stress and forest fires leading to air contamination and respiratory problems that put pressure on healthcare systems. The need of excessive energy for air conditioning can result in black-outs due collapsing grids and put further strain on societies and healthcare systems. In such a case scenario planning faces the difficult mission to find energetically and psychologically feasible strategies that allow for the provisioning of clean air and optimal temperatures for potentially large communities during such extreme events.

 It is clear that crises provide learning opportunities that can be translated into scenario planning. For instance, in some countries employment models with limited access to social security of care givers, very often with an immigrant background, have proven disastrous in terms of fatalities in geriatric asylums due to the introduction and spread of COVID-19. Based on such outcomes, change in contract models that provide full access to social benefits during (preventative) sick leaves of such care givers would have substantial positive outcomes for survival. This example indicates, however, that best-case scenarios can still incur substantial costs for health systems: for instance, in terms of potentially high (governmental) costs for providing equal social benefits across populations.

 Also, scenarios should not be seen as static endpoints and therefore need to be refined iteratively,^20^ given potential social-ecological realities that may have made scenarios obsolete (eg, future climate scenarios likely changing from less ideal to worst) or others emerged from the unknown unknowns through interdisciplinary collaborations (eg, new technological solutions). Scenario planning may be ultimately facilitated by big data and computer science, including artificial intelligence and machine learning approaches, which become more powerful with technological development and the accumulation of social, technological, environmental and economic data.^17^

 We conclude with acknowledging that our examples are not exhaustive but they shall demonstrate the overarching complexity that is necessary to consider for sound scenario planning for transformative change. Dedicated institutes for transdisciplinary approaches to resilience, such as The Lyda Hill Institute for Human Resilience (University of Colorado), are necessary to advance this agenda. Convergence Mental Health together with other transdisciplinary paradigms such as One Health and Brain Capital may inspire science-informed transformation in RiH and social-ecological systems. These and other transdisciplinary paradigms can further inform RiH planning and management through alternative, provocative and unorthodox approaches that are so far not considered worth of funding under current models of basic and applied research and their translation into practice, but which may be promising^16^: for example, Eastern mysticism, spirituality, religion, science fiction, the arts and quantum mechanics.

## Ethical issues

 Not applicable.

## Competing interests

 Authors declare that they have no competing interests.

## Authors’ contributions

 DGA conceived and wrote the paper. All coauthors have contributed to idea development and the writing.
